# Encyclopædia Medica

**Published:** 1899-12

**Authors:** 


**Encyclopædia Medica.** Under the General Editorship of Chalmers Watson, M.B. Vol. I.—*Abdomen to Bone.* Pp. vi., 579. Edinburgh: William Green & Sons. 1899.

The fifty articles of this volume are written by almost as many well-known physicians and surgeons of Edinburgh, Manchester, London, Birmingham, Bristol, Aberdeen, Leeds, Dundee, Dublin, Rome, Strathpeffer, and Montreal. The whole work is intended to be a complete and authoritative library, covering the entire field of general medicine and surgery, midwifery, diseases of children, eye, ear, and throat, public health, tropical diseases, &c., and it is expected that this encyclopædia "will become the generally accepted work of general reference by the profession." These are the days of encyclopædias: it is difficult for the busy practitioner to form a complete reference library in any other way than by providing himself with some such work as this or a combination of them.

There is neither introduction nor preface to this volume, but we learn from a circular which was issued before the publication of the work that it will be complete in twelve volumes, which will be published quarterly, and "it is intended to keep it up to date by the issue from time to time, and at a small cost, of a supplementary volume, and along with it a simple and original device in the shape of gummed printed marginal notes."

As regards the articles themselves, it is difficult to select any for especial criticism: there must be some inequality as regards the different subjects—*e.g.*, the whole subject of antipyretics is dismissed in about three pages, whereas blackwater fever occupies ten. Anthelmintics are dismissed in less than a page, and chloroform is not mentioned as a vermifuge. The article on Balneology, by Dr. Fortescue Fox, is very full, comprising some twenty-seven pages, but we are not surprised to observe that our local Spa has not yet earned a right to the distinction of a reference. The articles on Alcohol, Alcoholic Insanity, and Alcoholism are full ones. They are written by Dr. William Ewart and Dr. George Wilson; they extend to thirty-one pages, and are an excellent epitome of the use and abuse of alcohol in health and disease. Mr. Stanley Boyd gives thirty-two pages on Aneurysm, and Dr. Dreschfeld an additional seven pages on Abdominal Aneurysm; a third article on Aneurysm and Dilatation of the Thoracic Aorta, by Dr. Graham Steell, extends to ten pages, so that we have in all nearly fifty pages on the important subject of aneurysm, which, much to our surpise, is not elucidated by a single diagram or illustration of any kind. The article on the Physiology and Clinical Investigation of Blood is by Dr. T. H. Milroy, who gives a condensed epitome in twenty pages of everything needful to the understanding of the varieties of anæmia described by Dr. G. Lovell Gulland.

Dr. James Swain writes on Injuries of the Abdomen, and deals in an interesting way with injuries to the various abdominal viscera and the methods of treating such injuries. He also writes on Traumatic Peritonitis and on Abdominal Abscess.

The important subject of Anæsthesia is considered in four sections. Dr. Dudley Buxton writes on the Physiology of Anæsthetics, and on Minor Anæsthetics, while Chloroform and Ether are written on respectively by Dr. Alexander Ogston and Mr. Pridgin Teale; the latter writes with all his well-known enthusiasm on the subject of ether and its superiority over chloroform, but he apparently still uses it without the preliminary administration of nitrous oxide, for he makes no mention of this, but refers the reader to the chapter on Minor Anæsthetics for any account of this now universal preliminary to the administration of ether.

The article on Appendix Vermiformis is by Mr. F. J. Shepherd, of Montreal, who deals with the subject of appendicitis in an eminently judicial spirit. He says very truly that in acute cases it is always better to operate if one is in doubt, for early operations are comparatively safe, and he has never repented having operated too early, but has been sorry in many cases that operation had been postponed until too late.

The article on Auditory Nerve and Labyrinth is by Dr. Dundas Grant, who writes with his usual clearness on the various tests for nerve-deafness, as well as on the different varieties of the affection. The first volume ends with an important chapter on Diseases of Bone, by Mr. Alexis Thomson, who gives an excellent account of the bacterial forms of bone-disease, such as tubercle and syphilis, and a brief account of the tumours of bone; other diseases of bone, such as rickets, scurvy, &c., are to be dealt with under their special headings.

Dr. Chalmers Watson is to be congratulated on having secured the help of so many eminent men in his colossal undertaking. The "literature" appended to some of the articles strikes us as meagre. The book is very sparsely illustrated. Achondroplasia has one not very informing process block; there are three illustrations of anæsthetic apparatus; a diagram in each of the articles on Antipyretics and Aphasia; some small woodcuts illustrating ligature of arteries and artificial limbs; an excellent plate of urinary calculi, and a coloured picture of normal blood.

